# Kondo effect and superconductivity in niobium with iron impurities

**DOI:** 10.1038/s41598-021-93731-6

**Published:** 2021-07-09

**Authors:** Hansong Zeng, Dan Zhou, Guoqing Liang, Rujun Tang, Zhi H. Hang, Zhiwei Hu, Zixi Pei, X. S. Ling

**Affiliations:** 1 Institute for Advanced Study, School of Physical Science and Technology, Soochow University, Suzhou, 215006 People’s Republic of China; 2 Institute of Physics, Chinese Academy of Science, Beijing, 100190 People’s Republic of China; 3 Department of Physics, Brown University, Providence RI, 02912 USA

**Keywords:** Physics, Condensed-matter physics, Superconducting properties and materials

## Abstract

Kondo effect is an interesting phenomenon in quantum many-body physics. Niobium (Nb) is a conventional superconductor important for many superconducting device applications. It was long thought that the Kondo effect cannot be observed in Nb because the magnetic moment of a magnetic impurity, e.g. iron (Fe), would have been quenched in Nb. Here we report an observation of the Kondo effect in a Nb thin film structure. We found that by co-annealing Nb films with Fe in Argon gas at above 400 $^{\circ }$C for an hour, one can induce a Kondo effect in Nb. The Kondo effect is more pronounced at higher annealing temperature. The temperature dependence of the resistance suggests existence of remnant superconductivity at low temperatures even though the system never becomes superconducting. We find that the Hamann theory for the Kondo resistivity gives a satisfactory fitting to the result. The Hamann analysis gives a Kondo temperature for this Nb–Fe system at $\sim$ 16 K, well above the superconducting transition onset temperature 9 K of the starting Nb film, suggesting that the screening of the impurity spins is effective to allow Cooper pairs to form at low temperatures. We suggest that the mechanism by which the Fe impurities retain partially their magnetic moment is that they are located at the grain boundaries, not fully dissolved into the bcc lattice of Nb.

## Introduction

Effects of impurities on superconductivity have been of longstanding interest in quantum condensed-matter physics^1^. According to Anderson’s theorem^2^, nonmagnetic impurities should have little effect on the superconducting transition temperature $T_{\mathrm{c}}$. Magnetic impurities cause spin-flip scattering of the conduction electrons thus will have a large effect on the Cooper pairing in a superconductor, i.e. suppressing $T_{\mathrm{c}}$, as indeed observed^3^. However, a striking exception^4^ was that dilute amount of Fe dissolved in Nb seems to have little effect on the superconducting transition temperature, and magnetic susceptibility measurement showed^3,4^ that the magnetic moment of Fe was absent in the host metal Nb. Anderson explained^5^ that whether a magnetic impurity ion can retain its magnetic moment depends on the density of states at the Fermi energy $E_F$ of the host metal. The Anderson model explains nicely why Fe in molybdenum is magnetic and can produce a Kondo effect, while Fe in Nb cannot, since Nb has a larger density of state at $E_F$ than molybdenum^6^. Thus what we are reporting here came first as a surprise that when we introduced a very dilute amount of Fe into a Nb thin film, we observed a drastic reduction in $T_{\mathrm{c}}$ and a well developed Kondo effect. Details of our observation will be described below.

The Kondo effect refers to an anomalous minimum in the temperature dependence of resistivity in a metal first observed in gold wires^7^ and later Kondo^8^ discovered that the anomalous increase of resistivity with decreasing temperature can arise from a strong (divergent) spin-flip scattering between the magnetic impurities and the conducting electrons of the host metal when their exchange interaction is antiferromagnetic. Later improved theories^9–11^ showed that with decreasing temperature, the spin-flip scattering on the conduction electrons due to the localized spin on magnetic impurities do indeed increase with decreasing temperature, however, there is no divergence at *T* = 0. Instead, the localized spin and the conduction electron spins form a virtual many-body bound state below a characteristic temperature $T_{\mathrm{K}}$. At *T* = 0 the localized spin is completely screened. There is no divergence in resistivity as *T* approaches absolute zero. Further theoretical improvements were also provided to account for the orbital angular momentum of the impurity atoms^12^.

Early experimental studies of Kondo effect were on metals^7,13–17^, recently there have been reports of Kondo effect in materials like oxide films and carbon-based materials^18–21^, and most recently in transition metal dichalcogenides^22^. Various methods have been used to introduce magnetic impurities into the host material, such as chemical vapor transport method, melt growth method and paramagnetic gating, etc.^23–27^.

Superconductivity emerges from another nontrivial bound state in a many-body quantum system: Cooper’s pairing^28^ of two electrons with opposite spins and momenta on the Fermi surface mediated by the electron–phonon interactions. The formation of a phase coherent ground state of many Cooper pairs results in resistance vanishing when the temperature is lowered to below $T_{\mathrm{c}}$^29^. When magnetic impurities are incorporated into the system, the interplay between these two competing ground states has been a subject of great interest^1,30–35^. In superconducting alloys, Kondo effect can change its superconducting properties greatly^36–38^. The competition between superconductivity and Kondo effect has been of great interests^39–42^. In recent years, the Kondo effect has also been studied in many new superconductors materials^31–35^, in 2D topological superconductors and 3D topological insulators^43,44^. Similar resisivity minimum was also observed in high-$T_{\mathrm{c}}$ superconductors^45^.

Nb is an important superconducting material for its stability and for having the highest superconducting transition temperature (9.2 K) among the elemental metals. Superconducting properties of Nb have been investigated intensely over the years^46–51^ and in theories^52,53^. Thin films of Nb are of great interest for model systems^54^ and devices^55^. NbSe$_2$ is a close relative of Nb, a recent experiment^56^ revealed that superconductivity and magnetism can coexist in NbSe$_2$. Thus it is of great interest to explore if one can create Kondo effect in Nb as well in spite of early studies^3,4,6^ that suggested the quenching of Fe magnetic moment in Nb. Here we report transport measurements of sputtered Nb thin films containing low density of Fe impurities that clearly demonstrate the existence of the Kondo effect in a Nb thin film structure.

## Results and discussion

### The effects of co-annealing with Fe

**Figure 1:**
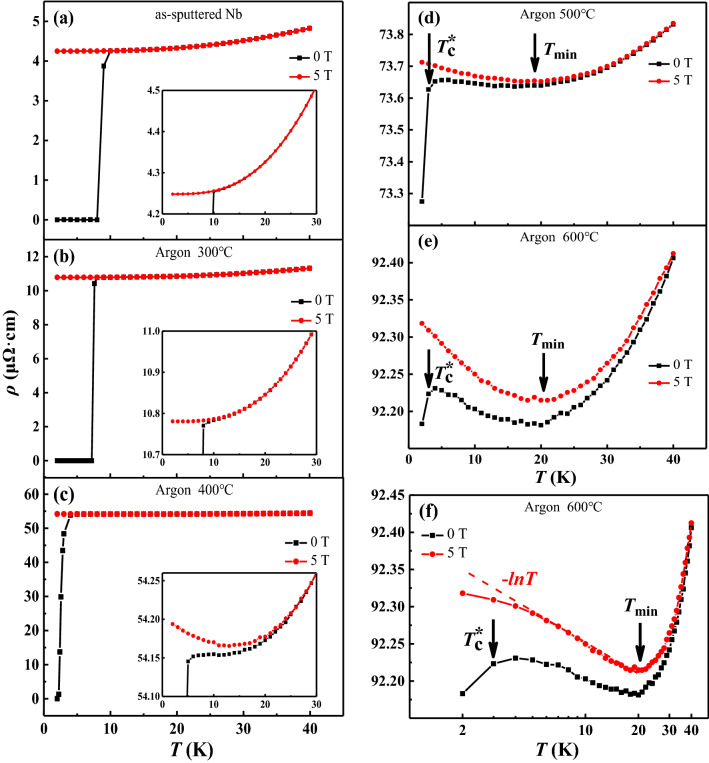
The effects of co-annealing with Fe in argon gas: (**a**–**e**) are the temperature dependence of resistivity $\rho$ vs. *T* at 0 T and 5 T for as-sputtered Nb film, annealed at 300 $^{\circ }$C, 400 $^{\circ }$C, 500 $^{\circ }$C and 600 $^{\circ }$C, respectively. The annealing was done in flowing argon gas (pressure 50 Pa), for 60 min; (**f**) The data in (**e**) are re-plotted in a semi-log plot, with the *x*-axis being in logarithmic scale. The regime of $-lnT$ is marked by a dashed straight line. The onset of superconducting transition $T_{\mathrm{c}}^*$ is defined as the first drop of $\rho$.

Figure 1 shows the main findings of this work. Figure 1a shows the temperature dependence of the resistivity of an as-sputtered sample in zero field and at 5 T. The superconducting transition temperature $T_{\mathrm{c}}$ is defined as the temperature at which the sample resistivity has dropped to 1/2 of its normal state value (at 10 K). The onset temperature of superconductivity $T_{\mathrm{c}}^*$ is defined as the data point where the resistivity shows a clear drop. The as-sputtered sample shows $T_{\mathrm{c}}$ = 8.875 K in zero magnetic field. At 5 T, the $\rho$–*T* is a smooth monotonic function of temperature down to 2.0 K.

**Figure 2:**
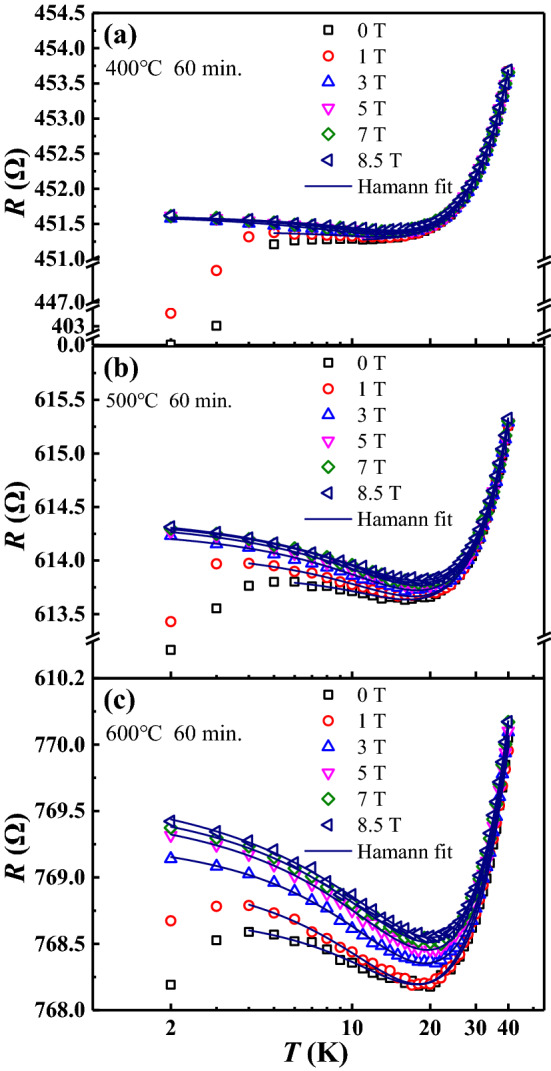
The effects of magnetic field on the *R*–*T* for Nb samples co-annealed at (**a**) 400 $^{\circ }$C, (**b**) 500 $^{\circ }$C, and (**c**) 600 $^{\circ }$C. The annealing time is 60 min. for all. The magnetic field ranges from 0 to 8.5 T. The room-temperature resistance *R* for the three samples: (**a**) 532.9 $\Omega$, (**b**) 688.1 $\Omega$ and (**c**) 861.7 $\Omega$, respectively. The solid lines are the fitting curves for the Hamann theory. Note that the *x*-axes are in logarithmic scale.

Figure 1b shows the temperature dependence of the resistivity of the sample annealed at 300 $^{\circ }$C, also in zero field and at 5 T. Immediately one notices the normal state resistivity is increased and the superconducting transition $T_{\mathrm{c}}$ is reduced. Yet, the $\rho$–*T* at 5 T behaves very similar to that of the as-sputtered sample in Fig. 2a. The $\rho$–*T* curve at 5 T is also a monotonic function of temperature.

Figure 1c–e show the $\rho$–*T* curves (at 0 T and 5 T) for the samples annealed at 400 $^{\circ }$C, 500 $^{\circ }$C, and 600 $^{\circ }$C, respectively. Two distinct features are immediately obvious to the eyes: there is a minimum in the $\rho$–*T* in zero field and at 5 T, and the sharp zero-field superconducting transition is replaced by a gradual decrease in resistivity. At 5 T, the $\rho$–*T* curves show a minimum around 20 K. Between 5 and 20 K, the $\rho$–*T* curves show the characteristic logarithmic dependence o temperature often found in Kondo effect systems^7,8^. In Fig. 1f, the data in Fig. 1e are re-plotted in a semi-log plot, with *x*-axis being logarithmic. The regime of $-lnT$ is marked by a dashed straightline, indicating the logarithmic behavior characteristic of the Kondo effect.

### Understanding the “normal” behavior

In Fig. 1a,b, the temperature dependence of the resistivity in a magnetic field of 5 T, when the superconductivity in the system is suppressed, behave as expected for “pure” Nb films. With decreasing temperature, the resistivity decreases monotonically towards a constant value. We made an attempt to fit these two data sets to the normal metal resistivity formula^57^ $\rho (T) = \rho _0+aT^2+bT^5$ that contains the residual resistivity (constant $\rho _{\mathrm{0}}$), the Fermi liquid contribution (electron–electron scattering, $\sim T^2$), and the electron–phonon interaction ($\sim T^5$). The fitting (not shown) is reasonably well considering the fact that it did not consider the effects of grain boundary scattering^58^ and surface scattering^59^. In the early study of the normal state resistivity of Nb^48^, it was argued that there should be a $T^3$ term in the $\rho$–*T* due to the inter-band scattering (Nb conduction band consists of a mixture of *s*–*d* bands^53^), in addition to the three terms discussed above. We found that by adding a $T^3$ term can indeed improve on the data fitting to almost perfection, however, the fitting parameters for the coefficients for the Fermi liquid $T^2$ and the electron–phonon $T^5$ terms become negative, *i.e.* nonphysical. Thus we believe the standard three terms are adequate description of the normal state resistivity in our as-sputtered and 300 $^{\circ }$C annealed Nb films.

Figure 1b deserves some special attention. After co-annealing at 300 $^{\circ }$C, the $T_{\mathrm{c}}$ of the Nb film is reduced slightly, by 1.4 K, yet there is no Kondo effect even at 5 T magnetic field when the superconductivity is fully suppressed. We believe this $T_{\mathrm{c}}$ reduction is the formation of NbO due to the substrate SiO$_2$, as also found previously^60^. NbO is a metal which becomes superconducting at 1.2 K^61^. Thus it is likely the layer of Nb that is in intimate contact with SiO$_2$ layer is converted to NbO which then reduces the $T_{\mathrm{c}}$ of the top layer of Nb by the proximity effect^62^. It should be noted that NbO is itself an interesting quantum material^63,64^.

### Ruling out localization

In disordered metals, in addition to the Kondo effect, there is another mechanism that can give rise to an anomalous increase in resistance as a function of decreasing temperature, namely the weak localization (for a review , see^65^). At very low temperatures, the quantum interference between the elastic scattering paths in a disordered metal can cause additional resistance. This resistance decreases with increasing temperature, before the eventual rise again in resistance due to the electron–phonon scattering. This weak localization effect can also lead to a resistance minimum without any magnetic impurities. We believe this is not what happens in our samples. For weak localization, adding magnetic field will suppress the quantum interference effects, resulting in a negative magneto-resistance^66^. Here, as shown in Fig. 2, the magneto-resistance below 30 K is positive for all samples.

As shown in Fig. 2, the *R* vs. *T* curves at different magnetic fields are shown, for the samples annealed at (a) 400 $^{\circ }$C; (b) 500 $^{\circ }$C; (c) 600 $^{\circ }$C. They all clearly show a resistance minimum around a $T_{\mathrm{min}}\sim$ 10–20 K. We also tried annealing in shorter time, 30 min., the effects are equivalent to that at lower temperatures for a longer time. For samples annealed at 400 $^{\circ }$C, 500 $^{\circ }$C and 600 $^{\circ }$C, the resistance minima $T_{\mathrm{min}}$ appear at $\sim$ 14 K, 16 K and 20 K, respectively. In Kondo’s model^8^, with decreasing temperature, the resistance minimum is where the scattering from the magnetic impurities start to dominate in the electron transport (we should point out that the charge carriers are hole-like in Nb due to the anisotropic shape of its band structure^67^).

Below $T_{\mathrm{min}}$, in zero magnetic field, the resistance increases with decreasing temperature, then reaches a maximum and starts to decrease again. For the 400 $^{\circ }$C-annealed sample, the resistance drops to zero near 2 K, while for 500 $^{\circ }$C and 600 $^{\circ }$C-annealed samples, the resistance remains at a large value down to 2 K. Nevertheless, we attribute the low-*T* decrease in resistance to the onset of superconductivity in the sample, or more precisely small regions of the sample becoming superconducting, even though the system as a whole does not have a percolating path of superconducting regions. This picture is confirmed when we apply magnetic field. As shown in Fig. 2a–c, at field larger than 3 T, the resistance increases with decreasing temperature to approach a saturation. The superconductivity is completely destroyed.

### Choosing theoretical framework for data analysis: the Hamann formula

To analyze the *R*–*T* curves in Fig. 2, we need a theoretical framework for the data analysis. In the original Kondo model^8^, the resistivity has a logarithmic dependence on temperature, or −ln(*T*/$T_{\mathrm{K}}$), where $T_{\mathrm{K}}$ is the Kondo temperature. In Fig. 2 we plot the resistance vs. log(*T*). There is indeed a region below $T_{\mathrm{min}}$ that the *R*(*T*) curves have a logarithmic dependence on temperature. Upon further decrease in temperature, however, the resistance levels off. This feature was not explained by Kondo^8^. It was later understood that the logarithmic divergence as *T* approaches absolute zero in Kondo’s model is avoided by a screening effect of the impurity spin by a cloud of antiparallel spins from the conduction electrons, due to their antiferromagnetic exchange interactions^9,11,68^. This occurs below $T_{\mathrm{K}}$. The screening of the impurity spin by the Kondo cloud^69^ leads to a levelling off in the scattering rate (hence resistivity) as *T* approaches absolute zero.

For analyzing experimental results on temperature-dependent resistivity, however, researchers need an analytical formula. The resistivity formula given by Hamann^11^ was the closest in describing experimental results^15^. In early experiments people adopted an empirical approach to mimic theoretical results of complex forms^13,17^. Recently, using empirical approach for the numerical renormalization group (NRG) result has gained wide popularity^70–74^. We also attempted to use this empirical NRG approach to fit our data in Fig. 2. However, we found that there is an underlying inconsistency in the analysis: the extracted $T_{\mathrm{K}}$ is much larger than $T_{\mathrm{min}}$, where the resistance clearly deviates from the normal metallic behavior (Drude + Fermi liquid + electron–phonon scattering).

Thus we decide to follow a recent study of the Kondo effect in VSe$_2$^75^ and adopt the resistivity formula derived by Hamann^11^ using the theoretical approach proposed by Nagaoka^10^,

1 $$\begin{aligned} R(T) =R_0+q{T^2}+p{T^5}+R_1(1-\frac{{\ln (\frac{T}{T_{\mathrm{K}}}})}{[\ln ^2(\frac{T}{T_{\mathrm{K}}})+S(S+1)\pi ^2]^{1/2}}), \end{aligned}$$

where $R_{\mathrm{0}}$ (Drude), *q* (Fermi liquid), *p* (electron–phonon), $R_{\mathrm{1}}$ (Hamann’s unitarity limit), $T_{\mathrm{K}}$ (Kondo) are fitting parameters.

The fitting curves using Eq. 1 are shown in Fig. 2 overlaying on top of the experimental data points. The agreement is striking. We found that if we set the impurity spin *S* = 1/2 or larger, the fitting routine cannot converge. This was known to the field^16,22^ for quite sometime, and perhaps the main motivation for the empirical approaches^13,17,22,70^. Thus we also kept *S* as a fitting parameter. We find that the extracted *S* = 0.1–0.17, the extracted Kondo temperature $T_{\mathrm{K}}$ = 15–24 K, for magnetic field = 0–8.5 T.

### Kondo temperature $T_{\mathrm{K}}$ and $T_{\mathrm{min}}$

**Figure 3:**
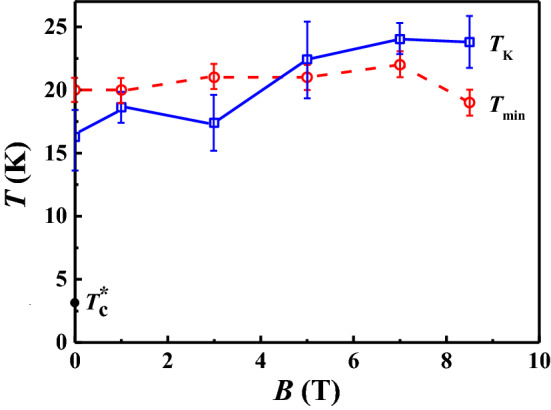
Characteristic temperatures vs. magnetic field $\mathbf {B}$. $T_{\mathrm{K}}$ is the extracted Kondo temperature from Fig. 2 using the Hamann theory. $T_{\mathrm{min}}$ is the temperature where the resistance vs. temperature curve has a minimum, $T_{\mathrm{c}}^*$ is the zero-field onset temperature for superconductivity. All are deduced from Fig. 2c.

In Fig. 3, we compare the Kondo temperature with the resistance minimum temperature $T_{\mathrm{min}}$. The extracted Kondo temperatures $T_{\mathrm{K}}$ are consistent with the physical picture of Kondo^8^ that the spin-flip scattering starts to dominate at temperatures around the resistance minimum. In comparison, the Kondo temperatures $T_{\mathrm{K}}$ extracted using the empirical NRG approach are much larger than $T_{\mathrm{min}}$. In comparison, the remnant superconductivity sets in at $T_{\mathrm{c}}^*$ = 3 K. This is consistent with the expectations that the localized spin on Fe is sufficiently screened that the electron–phonon interaction is strong enough to create localized, non-coherent, superconducting regions.

**Figure 4:**
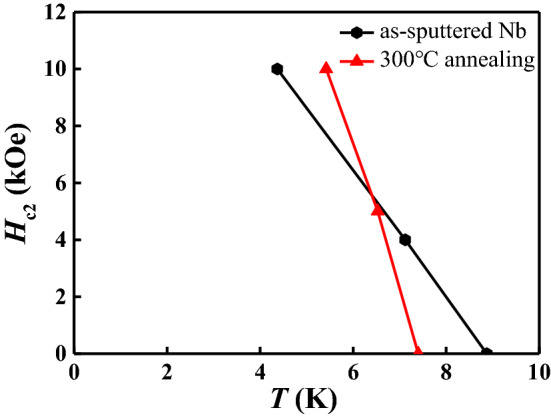
The upper critical fields $H_{c2}(T)$ lines for the as-sputtered Nb and the 300 $^{\circ }$C co-annealed Nb films. The superconducting transition temperature $T_{\mathrm{c}}$ is defined at $\rho (T_{\mathrm{c}})$ = $\rho_{\mathrm{n}}$ (10 K)/2. The critical temperature $T_{\mathrm{c}}$ (0) of the as-sputtered Nb film is 8.875 K, and the superconducting transition width is about 52 mK (10–90$\%$ $R_{\mathrm{n}}$ criterion).

### Coherence length vs. mean free path

To gain some insights into the nature of the electronic states in the as-sputtered and 300 $^{\circ }$C annealed Nb films, i.e. the “normal” films, we measured the $H_{\mathrm{c2}}$–*T* phase diagrams near $T_{\mathrm{c}}$ for these two samples, as shown in Fig. 4. The $H_{\mathrm{c2}}(T)$ curves show linear temperature dependence near $T_{\mathrm{c}}$, in agreement with the Ginzburg–Landau (GL) theory^62^. From the slopes of the $H_{\mathrm{c2}}(T)$ curves, using the GL theory we estimate $\xi$ to be about 12.9 nm for the as-sputtered sample, and 6.2 nm for the annealed at 300 $^{\circ }$C sample. We also estimated the Drude mean free paths *l* = ${{\hbar }{k_F}}/{ne^2{\rho _{xx}}}$ using their 10 K resistivities, $l\sim$ 18.5 nm for the as-sputtered, and 7.2 nm for the annealed at 300 $^{\circ }$C samples, respectively. These results suggest that the coherence length is of the same order of magnitude as the mean free paths. Since $1/\xi =1/\xi _0+1/l$, where ${\xi }_{\mathrm{0}}$ is the intrinsic coherence length, *l* is the mean free path, we conclude that the superconducting coherence length in our Nb films is determined by the electronic mean free paths. In Table 1, we summarize some of the key parameters of the five samples discussed in Fig. 1.

### Why is the magnetic moment of Fe not quenched in Nb thin films?

It is well-known that the magnetic moment of Fe impurities will be quenched when the Fe atoms are dissolved in Nb^3,4,6^. In those systems, mixing of Fe into Nb was done at 1200 $^{\circ }$C for a time period of a week. It is expected that Fe atoms are well incorporated into the bcc lattice of Nb, even though their exact locations are unknown. Here, given the highest temperature we used, 600 $^{\circ }$C, the Fe atoms are expected to be adsorbed between the crystalline grains, i.e. not fully dissolved into the bcc lattice of the Nb crystalline grains. The fact that the extracted spin parameter $S\sim$ 0.10–0.17 is small is consistent with Anderson’s theory^5^ on local magnetic moment formation in metals.

### The sign of magneto-resistance

The sign of the magneto-resistance of the Kondo effect contribution should be negative since the magnetic field tends to destablize the virtual bound state between the localized moment and the conduction electrons. As shown in Fig. 2, the sign of magneto-resistance here is positive, the resistance increases with magnetic field. In the Au–Fe system^76^, the raw data of magneto-resistance was also positive, only after subtracting out the non-Kondo part can one see the negative magneto-resistance. Unfortunately, such a subtraction procedure cannot be carried out for the Nb–Fe system here due to the residual superconductivity in the system. As shown in Supplementary Fig. SI-2 (in Supplementary Information), we re-plot the data in Fig. 2c as a function of magnetic field for the Nb sample annealed at 600 $^{\circ }$C. In Supplementary Fig. SI-2a, below 10 K, there is a large positive magneto-resistance effect due to the destruction of the Cooper pairs with increasing field. Above 10 K, shown in Supplementary Fig. SI-2b, the magneto-resistance effect is small, but still positive. This is the well known “classical” magneto-resistance of metals due to grain boundaries and Fermi surface anisotropy as discussed by Giordano^76^. However, due to the presence of the residual superconductivity in the Nb–Fe system here, the subtraction procedure of Giordano^76^ cannot be applied. Thus we rely on the curve fitting using Hamann’s theory^11^.

## Conclusions

We report a resistance anomaly of sputtered Nb films after co-annealing with Fe in inert gas at different temperatures up to 600 $^{\circ }$C. We found that with an increase of annealing temperature from 300 to 600 $^{\circ }$C, the superconducting transition temperature ($T_{\mathrm{c}}$) of Nb film changes sharply from 8.9 K to below 2 K, even though there are hints of superconductivity remaining below 3 K for samples annealed at 600 $^{\circ }$C for 60 min. Moreover, for films annealed at above 400 $^{\circ }$C, a *R*–*T* resistance minimum is observed which persists under different magnetic field up to 8.5 T. This resistance minimum can be well fitted with the Hamann resistivity formula. We suggest that the survival of the magnetic moment of Fe impurities in Nb is due to their not being fully dissolved into the bcc structure of Nb.

In spite of the strong agreement with the Hamann theory we found in this report, a few words of caution are warranted. First, we do not have direct evidence that the Fe impurity atoms retain their magnetic moments, a direct test (e.g. using spin-polarized STM) would be highly desirable. Second, the formation of the Kondo bound states may be detectable in the change of effective mass of the charge carriers.

**Table 1:** Summary of the parameters measured from the as-sputtered Nb film and the annealed samples.

Annealing temperature ($^{\circ }$C)	$T_{\mathrm{c}}$ (K)	$T_{\mathrm{c}}^*$ (K)	$\rho$ ($\upmu \Omega$ cm) (10 K)	*l* (nm) (10 K)	$\xi$ (nm)
0 (as-sputtered Nb)	8.875	9	4.25	18.5	12.9
300	7.4	7.6	10.8	7.2	6.2
400	2.4	3	54.2	1.46	–
500	–	3	73.6	1.07	–
600	–	3	92.2	0.86	–

## Methods

The five samples used in this paper are from the same Nb thin film wafer. Our Nb film has a thickness of 120 nm, deposited on SiO$_2$/Si substrates by DC magnetron sputtering, as reported previously^77,78^. The SEM micrographs show that the films are granular in nature, with average grain size around 20 nm in as-sputtered sample and the grain sizes increase with annealing temperature, up to about 40 nm at 600 $^{\circ }$C, as shown in Supplementary Fig. SI-1 in the Supplementary Information. The Nb films were patterned into 4-probe devices, using standard photo lithography and ion beam etching. The sample region is a micro-bridge of sizes 1 mm $\times$ 10 $\upmu$m. The sample was annealed in the thermal annealing chamber filled with flowing argon gas (purity: 99.99$\%$, pressure: 50 Pa, flow rate: 20 sccm) at different temperatures (300 $^{\circ }$C, 400 $^{\circ }$C, 500 $^{\circ }$C and 600 $^{\circ }$C) and for different duration (30 min and 60 min). During the annealing, the Nb film chip was held using an iron clip. Even though the annealing temperature is far below the melting point (1538 $^{\circ }$C) for Fe, the bombardment by argon gas molecules was enough to transport a minute amount of Fe into the Nb film sample. This “co-annealing” method of introducing Fe impurities into a host metal is similar to that of a previous experiment^79^. After annealing, the Nb samples were naturally cooled to room temperature under half of an atmosphere pressure of argon gas.

The temperature dependent (2–100 K) resistances of the samples were measured by standard four-probe method with magnetic field perpendicular to the film surface using a commercial system, the Physical Property Measurement System (PPMS-9, Quantum Design Inc.). The resistances were measured with the AC resistance mode in PPMS. The ohmic resistance measurements were carried out using a current of 120 $\upmu$A.

An elemental analysis of the annealed samples using the Time of Flight Secondary Ion Mass Spectrometry (TOF-SIMS) technique was conducted by a commercial service (WinTech Nano, address provided in the caption of Supplementary Fig. SI-3). In this test, 10 nm of the Nb film was sputtered away from the surface of annealed sample before taking the TOF-SIMS measurements in order to get the internal information of the sample. For the Nb film annealed at 600 $^{\circ }$C for 60 min. in Ar atmosphere, the surface density of Fe atoms is about 7–10 $\times$ 10$^{-3}$ $\upmu$m$^{-2}$, see Supplementary Fig. SI-3. This is about 0.1 Fe atom per 1 $\upmu$m $\times$ 1 $\upmu$m area, or 1 Fe atom per 3.2 $\upmu$m $\times$ 3.2 $\upmu$m area. For the Nb films annealed at 500 $^{\circ }$C and lower, the TOF-SIMS measurements yielded Fe signals, but too scattered to be reliable, see Supplementary Fig. SI-4.

## Supplementary Information

Supplementary Figures.
