# Supplementary material for: Kondo effect and superconductivity in niobium with iron impurities
Source: Sci Rep. 2021 Jul 9;11:14256. doi: 10.1038/s41598-021-93731-6 (PMC8270948; doi:10.1038/s41598-021-93731-6)
Supplement: Supplementary file 1 — Supplementary Figures. [file 41598_2021_93731_MOESM1_ESM.pdf]

Supplementary Information: Kondo effect and superconductivity in niobium with iron impurities

Hansong Zeng[1], Dan Zhou[1], Guoqing Liang[1], Rujun Tang[1,*], Zhi H. Hang[1], Zhiwei Hu[2], Zixi Pei[2], and X. S. Ling[3,*]

[1]Institute for Advanced Study, School of Physical Science and Technology, Soochow University, Suzhou, 215006, P. R. China
[2]Institute of Physics, Chinese Academy of Science, Beijing, 100190, P. R. China
[3]Department of Physics, Brown University, Providence, Rhode Island 02912, USA
[*]tangrj@suda.edu.cn; xinsheng_ling@brown.edu

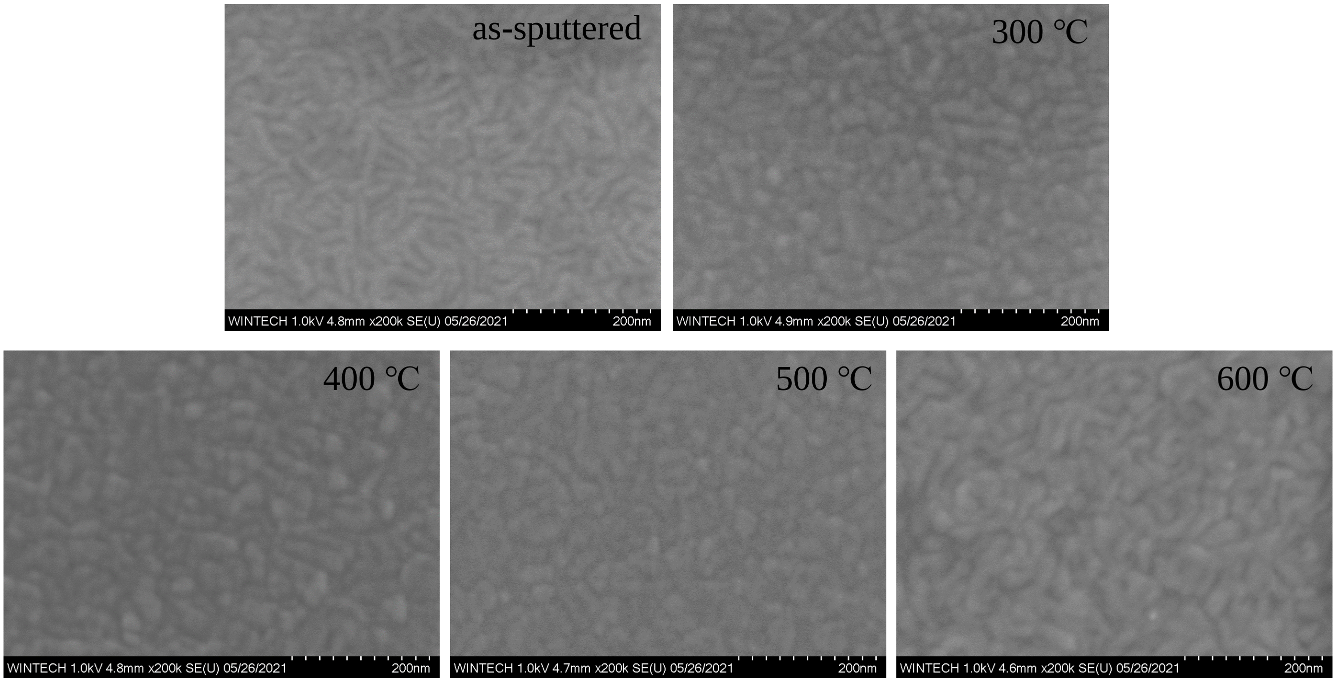

Figure SI-1. SEM micrographs of the Nb thin film samples discussed in the main text.

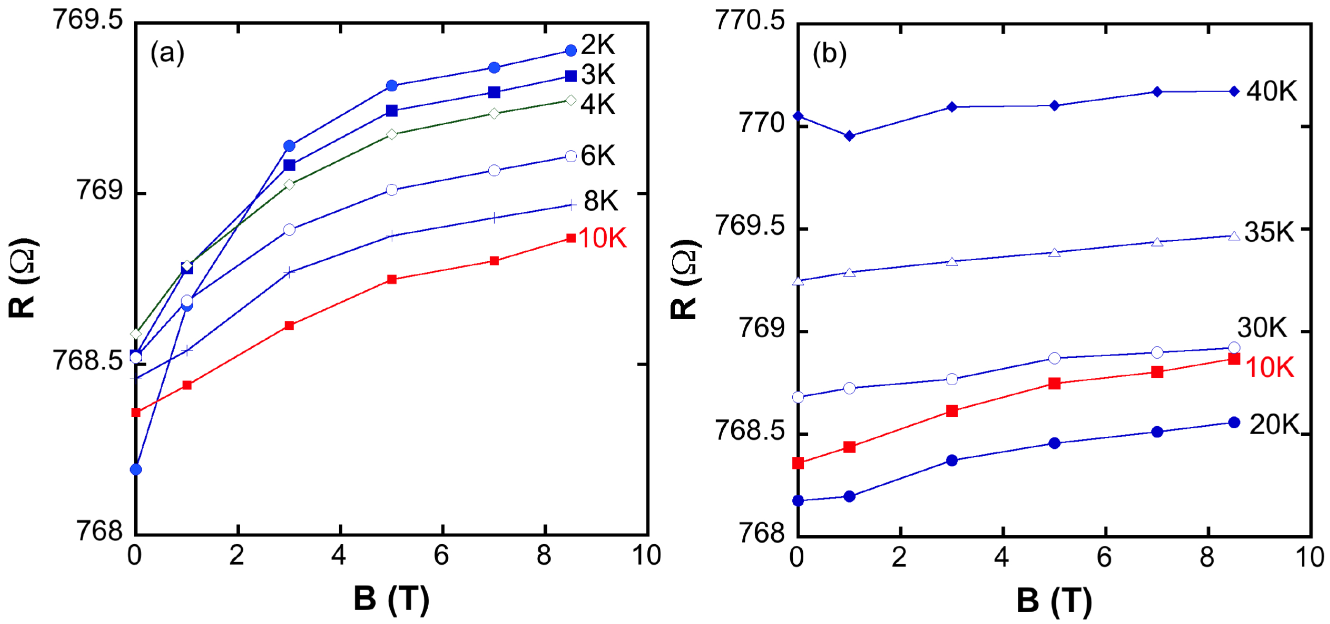

Figure SI-2. Magnetoresistance at different temperatures, re-plotted from Fig.2(c) of the main text.

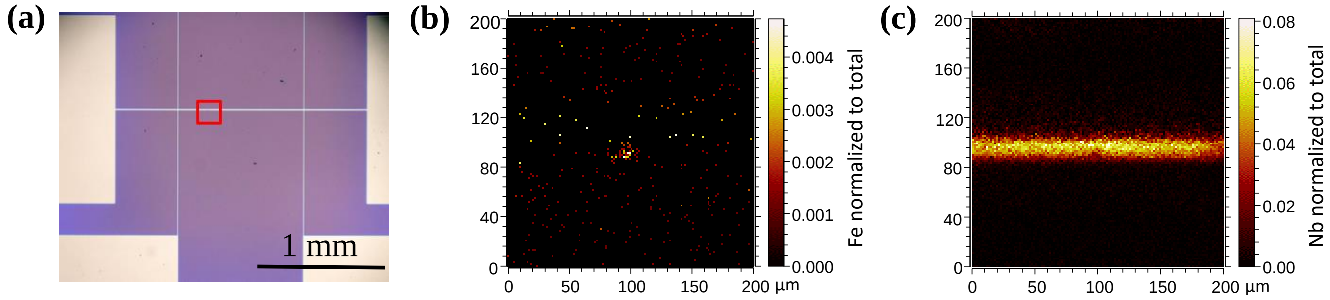

Figure SI-3. The TOF-SIMS analysis of the Nb film device annealed at 600°C for 60 min. in Ar gas. (a) The characterization area of 200 × 200 μm^2 is marked by the red square. The color map of the spatial distribution intensities (bright dots) of (b) Fe ions and (c) Nb ions. (The analysis was done by WinTech Nano-Technology Services Pte. Ltd., 10 Science Park Road, #03-26 The Alpha, Singapore Science Park II, Singapore 117684, www.wintech-nano.com)

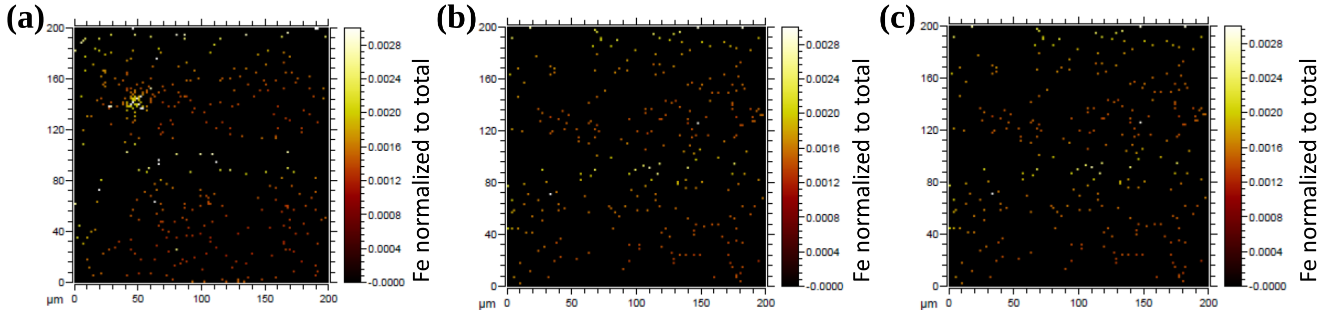

Figure SI-4. Fe signals in the TOF-SIMS analysis of the Nb film device annealed at (a) 300, (b) 400, (c) 500°C for 60 min. in Ar gas.
